# Born This Way? National Collective Narcissism, Implicit Homophobia, and Homosexual Essentialism in Populist Poland

**DOI:** 10.1007/s10508-024-02952-z

**Published:** 2024-08-16

**Authors:** Dorottya Lantos, Richard C. M. Mole, Agnieszka Golec de Zavala

**Affiliations:** 1 UTS Business School, University of Technology Sydney, Ultimo, 2007 Australia; 2 School of Slavonic and East European Studies, University College London, London, UK; 3 Department of Psychology, Goldsmiths, University of London, London, UK

**Keywords:** Implicit homophobia, National collective narcissism, Populism, Sexual orientation, Essentialism, Implicit Association Test

## Abstract

Prejudice toward the LGBT community has become prevalent in Poland under the ultraconservative populist government. The results of three studies conducted between 2018 and 2019 (*N*_*1*_ = 879, *N*_*2*_ = 324, and *N*_*3*_ = 374) indicate that Polish collective narcissism—the belief that the exaggerated greatness of the nation is not recognized by others—is associated with implicit homophobia assessed as the intuitive disapproval of gay men and automatic evaluative preference of heterosexuality over homosexuality. Those associations were to a large extent explained by the relationships between collective narcissism and (1) the belief that groups defined by sexual orientations are essentially distinct; (2) the belief that homosexuality is a personal choice, not genetically determined or culturally universal. The experimental results of Study 3 indicated that inducing the belief that non-normative sexuality is genetically determined and culturally universal reduced automatic preference for heterosexuality over homosexuality (but not intuitive disapproval of gay men) across levels of collective narcissism (contrary to predictions). The obtained results complete the picture of the association of narcissistic beliefs about the nation and homophobia emerging from previous studies. National narcissism is linked not only to explicit but also to latent, implicit homophobia likely to be triggered by increased presence of national narcissism in public discourse. Moreover, national narcissism is linked to implicit homophobia, especially via the agentic belief that sexual orientation is a matter of choice. Changing this belief reduces implicit homophobia also among national narcissists.

**Supplementary Information:**

The online version contains supplementary material available at 10.1007/s10508-024-02952-z.

## Introduction

Poland provides an intriguing context to study the relationship between the beliefs about national identity and attitudes toward sexual minorities (Mole et al., 2022). Poland is unique in Europe in never having criminalized homosexuality. No legislation banning homosexual activity between consenting adults was ever introduced in independent Poland, although such laws, common in Europe, were enacted on Polish territory by the occupying powers during the country’s partition (1795–1918) and remained in force until 1932. During the communist period, same-sex relations—while not illegal—were presented by the regime as “a symptom of ‘Western depravity’ and as inconsistent with ‘socialist morality’” (Kliszyński, 2001). Following the collapse of communism, life for LGBT Poles improved but same-sex rights remain limited. Poland is ranked bottom among all EU member-states in the ILGA-Europe Rainbow Index in terms of legal equality for queer citizens (ILGA-Europe, 2023)^1^ and lags far behind most other EU member-states in terms of social attitudes toward same-sex rights (Eurobarometer, 2019). These negative public attitudes have been fuelled by the propaganda and politics of the ultraconservative populist party *Law and Justice,* which came to power in 2015. Understanding the psychological predictors of homophobia in Poland has acquired topicality and urgency.^2^

In their attempts to limit the definition of the “people” in whose name they claim to speak and thereby delegitimize any voices that oppose their rhetoric and behavior, populist politicians in Poland have weaponized homophobia, with homosexuality presented as an “ideology” and “civilizational invasion” antagonistic to traditional family values rooted in the teachings of the Catholic Church (Santora, 2019). The LGBT community has been labeled a “rainbow plague” (Reuters, 2019) and in 2019 several cities declared themselves “LGBT free zones.”^3^ Participants of the first pride parade in the conservative Polish town of Bialystok were violently attacked by alt right activists (Santora, 2019). The COVID-19 pandemic intensified animosity toward the LGBT community, and the “anti-LGBT ideology” narrative was at the core of Andrzej Duda’s 2020 presidential campaign (Golec de Zavala et al., 2021a, 2021b; Walker, 2020). The largely uncontested rejection of the LGBT community in public life under *Law and Justice* suggests the existence of latent homophobia in Poland over and above the explicit, overt homophobia identified by national surveys and expressed in political discourse (Mole et al., 2022). In the present research, we examine whether latent, implicit homophobia is associated with the Polish national narcissism that characterizes supporters of *Law and Justice* (Golec de Zavala, 2023).

National collective narcissism is a belief that the exaggerated greatness of the nation is not sufficiently recognized by others (Golec de Zavala & Lantos, 2020; Golec de Zavala et al., 2009, 2019). It is a robust predictor of explicit prejudice toward stigmatized groups within the nation (e.g., women; Golec de Zavala et al., 2021a, 2021b; immigrants, ethnic minorities, Golec de Zavala et al., 2020). National collective narcissism has also been linked to overt and explicit homophobia in Poland (Golec de Zavala et al., 2021a, 2021b; Górska & Mikołajczak, 2015; Mole et al., 2022). However, research is yet to establish whether collective narcissism predicts latent and concealed, implicit prejudice. Implicit prejudice is an intuitive, automatic association of a targeted social group with a negative emotional reaction, not always available in introspection. Implicit and explicit prejudice are often unrelated as social norms may exist to prevent explicit expression of prejudice. It is unclear whether implicit and explicit prejudice are produced by the same or different cognitive processes (for a recent review and discussion, see Kurdi et al., 2023), but implicit and explicit prejudice tend to predict different behaviors (for a recent meta-analysis, see Kurdi et al., 2019). For example, implicit prejudice may be expressed in microaggressions—subtle, often unconscious derogations (Nadal et al., 2016). Implicit prejudice may also affect political decisions regardless of people’s conscious will to express prejudice (Cooley et al., 2014). Because of latent homophobia, people may become susceptible to political framing that misrepresents discrimination of the LGBT community as in-group defense (like framing the legalization of same-sex marriage as the victimization of its opponents, Jowett, 2017; Turner et al., 2018). Studies show that collective narcissism is related to endorsing such re-framing of prejudice (Cichocka et al., 2022) and a tendency to see the in-group as a victim rather than a perpetrator of discrimination (Golec De Zavala, 2022; West et al., 2022).

Seeking to extend the findings that homophobia is inspired by traditional beliefs about gender roles and homosexuality as a threat (Ayoub, 2014; Golebiowska, 2017), which are associated with national narcissism in Poland (Mole et al., 2022), we examine whether the relationship between Polish collective narcissism and implicit homophobia is mediated by essentialist beliefs about homosexuality, people’s lay theories that populists typically promote regarding the distinctiveness, immutability, and universality of homosexuality (Haslam & Levy, 2006; Rothbart & Taylor, 1992; Yzerbyt et al., 2001). Finally, we test whether inducing the essentializing “born this way” belief about sexual orientations reduces implicit homophobia, at least on low levels of collective narcissism.

### Polish Collective Narcissism and Homophobia

Like other forms of prejudice, homophobia is a function of normative beliefs maintained by societies, within which groups defined by non-normative sexual orientations are nested. Expressing prejudice that is supported and normative in a nation is one of the ways of declaring national identity (Crandall et al., 2002; Jost & Banaji, 1994; North & Fiske, 2014; Sidanius & Pratto, 1999). Beliefs justifying prejudice are accepted by members of advantaged (Lowery et al., 2006) and disadvantaged sub-groups within a nation (Dovidio et al., 2007, 2009), especially those high on national collective narcissism (Golec De Zavala, 2023; Golec de Zavala & Bierwiaczonek, 2021; Mole et al., 2022). National collective narcissism predicts prejudice over and above other robust predictors such as political conservatism, right wing authoritarianism, or social dominance orientation (Golec de Zavala, 2023, 2024; Golec de Zavala et al., 2019; Golec de Zavala & Lantos, 2020).

Previous studies suggest that Polish collective narcissists explicitly express homophobia to demonstrate their national and religious identity (Mole et al., 2022). However, prejudice is often ingrained in societal institutions and acquired during socialization as pervasive, self-perpetuating implicit bias. To the best of our knowledge, no previous research has examined the link between collective narcissism and implicit prejudice. In the present project, we attempted to answer the question as to whether Polish collective narcissism is linked to latent, implicit homophobia, an intuitive negative evaluation of homosexuality that goes beyond overt homophobia that serves to demonstrate social allegiance. We examine whether Polish collective narcissism is associated with two forms of latent homophobia: (1) the intuitive moral disapproval of gay men and (2) automatic preference for heterosexuality over homosexuality (Banse et al., 2001; Greenwald et al., 2002; Hatzenbuehler et al., 2009; Inbar et al., 2009; Rowatt et al., 2006). Implicit homophobia may be harbored regardless of whether people explicitly endorse it. It is important to investigate implicit bias because the awareness that it exists is the first step to down-regulate it.

Intuitive preferences and implicit biases are formed as a result of explicit propaganda (Arendt et al., 2015) and pervasive deflection and the re-framing of prejudice as protection of the in-group from victimization by malevolent others (Turner et al., 2018). We expected that people who endorse Polish collective narcissism would be susceptible to such a re-framing and ultimately to implicit homophobia. This is because they value in-group loyalty and follow the beliefs endorsed by group authorities and norms, especially when those beliefs justify intergroup hostility (Golec de Zavala, 2023, 2024). Collective narcissists are defensive, hypersensitive to intergroup threat and likely to endorse the reframing of discrimination as protection of the in-group (Golec de Zavala et al., 2016; 2022). Thus, we expect that the link between Polish collective narcissism and implicit homophobia will be driven by the specific beliefs about homosexuality emphasized by this propaganda (1) a non-essentialist belief that homosexuality is a matter of choice, a lifestyle, and an ideology and (2) an essentialist belief that non-normative sexuality is a social identity that is profoundly different to heterosexuality (Ayoub, 2014; Korolczuk & Graff, 2018; Mole et al., 2022). Those beliefs underpin homophobic propaganda in Poland hence our prediction that national narcissism will be positively associated with the belief in discreteness of social categories defined by sexual orientations but negatively associated with the beliefs in biological bases, immutability and universality of homosexuality.

### Beliefs Essentializing Homosexuality

Lay theories essentializing homosexuality argue that people defined by different sexual orientations constitute mutually exclusive groups. They possess distinct “essences” that profoundly differentiate them from each other and serve as intuitive bases for dispositional inferences about them (Haslam, 2017; Haslam et al., 2000; Yzerbyt et al., 2001). Social essentialism involves inappropriately treating social categories as distinct, universal, and unchangeable “natural kinds.” Essentialist beliefs predict prejudice in the case of some social groups (e.g., race; Mandalaywala et al., 2018), but the role of essentialist beliefs in predicting prejudice toward groups defined by sexual orientation is complex (Peretz-Lange, 2021).

Essentialist beliefs about homosexuality pertain to the discreteness of social categories defined by sexual orientations (i.e., a clear boundary differentiating homosexual or other non-normative sexualities from heterosexuals), their immutability (i.e., non-normative sexuality defined by predetermined, biological factors), and universality (i.e., their existence across all cultures and historical times; Haslam & Levy, 2006). Beliefs essentializing the difference between social categories defined by sexual orientations provide a structural explanation for homophobia. Biological determinism and cultural universality provide the “born this way” explanation of intergroup differences, discounting individual agency in choosing the stigmatized social identity (Peretz-Lange, 2021; for a similar idea in the aspect of "naturalness" of homosexuality, see Arseneau et al., 2013). While the belief in the discreteness of homosexuality predicts homophobia, the “born this way” beliefs in the immutability and universality of homosexuality predict tolerance and acceptance (Haslam & Levy, 2006; Haslam et al., 2002; Hegarty & Pratto, 2001; Herek & Capitanio, 1995; Huic et al., 2018; Jayaratne et al., 2006; Whitley, 1990).^4^

To our knowledge, no previous work has tested how collective narcissism and beliefs about homosexuality are associated. Given the content of homophobic propaganda in Poland, we expect that Polish collective narcissism will be positively associated with the “structural,” discreteness belief about homosexuality and negatively associated with the “born this way” beliefs comprising immutability and universality beliefs. We expect that those beliefs will independently mediate the association between Polish collective narcissism and implicit homophobia. Research indirectly supporting those expectations suggests that political conservatives (which national narcissists often are) are more likely to endorse the structural belief in the discreteness of homosexuality, and less likely to endorse the “born this way” belief in the immutability of homosexuality than liberals (Hoyt et al., 2019). Moreover, while experimental manipulations building on the different essentialist beliefs showed promise in shifting participants’ attitudes toward those with non-normative sexualities (Fry et al., 2020), other findings indicate that such experimental manipulation was only successful in increasing self-identified liberals’ belief in the immutability of homosexuality, but not that of conservatives (Hoyt et al., 2019). Collective narcissism is an aspect of political conservatism in Poland that may drive its association with beliefs essentializing homosexuality (Golec De Zavala & Keenan, 2021; Golec de Zavala et al., 2021a, 2021b). We expected that collective narcissism may limit the effectiveness of the intervention to reduce homophobia also because extant studies indicate that in-group identification and political conservatism (both associated with national narcissism) limit the effectiveness of prejudice reducing interventions (e.g., Turner et al., 2020; but see Golec de Zavala et al., 2024 for an intervention reducing prejudice at high national narcissism).

### Overview

In cross-sectional Studies 1 and 2, we tested two hypotheses. We predicted that (H1) Polish collective narcissism is associated with implicit homophobia and that (H2) this relationship is independently mediated by the “structural,” discreteness belief about homosexuality (positively) and the “born this way,” immutability and universality beliefs about homosexuality (negatively). Study 1 re-analyzed previously published data (Mole et al., 2022). Study 2 relied on a novel dataset to replicate the findings of Study 1. To ensure that our findings generalize across different conceptualizations and operationalizations of implicit homophobia, we operationalized implicit homophobia as the intuitive disapproval of gay men (Inbar et al., 2009) and as the automatic negative evaluation of homosexuality and positive evaluation of heterosexuality (assessed by the Implicit Associations Test [IAT]; Greenwald et al., 1998, 2009; Hatzenbuehler et al., 2009; Jost, 2019). Such evaluations acquired via social learning are difficult to control consciously and often not available through introspection. In Study 3, we tested two additional hypotheses, predicting that (H3) an experimentally induced “born this way” beliefs reduce implicit homophobia, (H4) at least on low levels of collective narcissism.

In all analyses, we controlled for participants’ age and gender, established demographic predictors of homophobia (Herek, 1984). We also controlled for national in-group satisfaction (i.e., feeling proud of belonging to a valuable nation; Leach et al., 2008) to ensure that collective narcissism is a unique predictor of implicit homophobia in comparison with another form of the nation’s positive evaluation (Golec de Zavala, 2023; Golec de Zavala et al., 2019). Following the recommendations of Simmons et al. (2011), we first tested our hypotheses without and then with the covariates. The results of the analyses without covariates consistently follow the pattern of results reported in the manuscript unless otherwise noted. These results are presented in Supplementary Materials. The syntax for all analyses can be found on OSF along the datasets (https://osf.io/uzr94/). All analyses were performed on the data of participants who reported heterosexual orientation. Power analyses were conducted using G*Power (Faul et al., 2007, 2009) and MedPower (Kenny, 2017).

### Data Analytic Plan

Descriptive statistics and reliabilities are first inspected together with correlations among the key variables and covariates. We also test gender differences on the intuitive disapproval of gay men and IAT performance to justify the inclusion of gender as a covariate.

To test H1, predicting that collective narcissism is associated with implicit homophobia, we conduct two linear regressions. In the first linear regression, we enter collective narcissism as the predictor, the intuitive disapproval of gay men as the outcome, and controlled for in-group satisfaction, age, and gender. In the second linear regression, we enter the IAT’s *d*-scores as the outcome.

To test H2, predicting that Polish collective narcissism is associated with implicit homophobia indirectly via the discreteness belief and independently via the “born this way,” immutability and universality beliefs, we conduct two multiple mediation analyses. First, we enter Polish collective narcissism as the predictor, the intuitive disapproval of gay men as the outcome, and the beliefs in the immutability and universality of homosexuality and the belief in the discreteness of homosexuality as independent mediators. In the second model, we test H2 using the IAT’s *d*-scores as the outcome. We include in-group satisfaction, age, and gender as covariates in both models. We use PROCESS macro for SPSS (Model 4; Hayes, 2018) and ask for 10,000 bootstrapped samples.

In the experimental Study 3, we conduct independent samples t tests on the effects of the experimental manipulation on the beliefs in the immutability and universality of homosexuality and on the belief in the discreteness of homosexuality as manipulation checks. We anticipate that participants in the experimental condition should indicate significantly greater beliefs in immutability and universality than those allocated to the control condition, while the manipulation should not affect participants’ belief in the discreteness of homosexuality if the experimental manipulation worked as intended.

To test H3, predicting that experimentally induced “born this way” beliefs reduce implicit homophobia, we conduct two independent samples *t* tests, first with the intuitive disapproval of gay men as the dependent variable and next with the IAT assessed implicit homophobia as the dependent variable, and the experimental manipulation as the independent variable.

To test H4, predicting that the experimental manipulation should be effective at least on low levels of collective narcissism, we conduct two moderation analyses. We first enter the intuitive disapproval of gay men as the outcome, the research condition, Polish collective narcissism, and their interaction as predictors. We next conduct the same model entering IAT scores as the outcome variable. We include age, gender, and national in-group satisfaction as covariates in the model in both models.

## Study 1

In Study 1, we tested H1 and H2 using a previously analyzed dataset (Mole et al., 2022, Study 2). Only the measure of national collective narcissism overlaps with those included in the previously published analyses.

### Method

#### Power Analyses

We used G*Power to estimate the sample sizes sufficient to test H1 (Faul et al., 2007, 2009). We conservatively assumed the average effect size reported across social psychological studies (*r* = .21 transformed to *f*^*2*^ = .04; Richard et al., 2003), given the lack of research on the link between collective narcissism and implicit prejudice, and given the moderate average effect size for the association between collective narcissism and explicit prejudice (Golec de Zavala et al., 2019). The sample size estimation for a linear multiple regression with alpha level = .05, power = .80, and 4 predictors yielded a minimum required sample of 304 participants.

We used the MedPower software to estimate the sample size necessary to test H2 (Kenny, 2017). For the association between national collective narcissism and implicit homophobia, we used the same effect size as above (*r*_*c*_ = .21). We assumed the same effect size for the association between national collective narcissism and essentialist beliefs about homosexuality (which was more conservative than the association between political conservatism and those beliefs reported previously, *r* = .35, Hoyt et al., 2019), and for the association between essentialist beliefs and implicit homophobia (which was more conservative than the effect size indicated by previous studies examining associations between those beliefs and explicit homophobia, *r* = .37, Hoyt et al., 2019). The analysis indicated a minimum sample of 228 to test H2 with alpha level = .05 and power = .80.

#### Participants

A nationally representative sample of 988 Polish adults completed the online survey via the Ariadna Research Panel (https://www.panelariadna.pl/). We analyzed data from 879 participants who indicated heterosexual orientation (418 women, ages 19–84 years, *M* = 43.17, *SD* = 13.59). The survey contained four attention checks (e.g., “Please select Agree”). Participants who failed any of the checks were not allowed to continue and their responses were automatically deleted.

#### Procedure

Participants completed an online survey ostensibly exploring the association between personality and social attitudes. All scales and all items within the scales were presented in a separate random order for each participant. Unless otherwise indicated, all measures were assessed on a 7-point scale (1 = *completely disagree*, 7 = *completely agree*).

#### Measures

*Collective narcissism* was measured with the Polish version of the 5-item Collective Narcissism Scale (Golec de Zavala et al., 2009; e.g., “I will not be satisfied until the Polish nation obtains the respect it deserves”), where higher scores indicate higher collective narcissism.

Essentialist beliefs about homosexuality were measured using a 15-item scale (Haslam & Levy, 2006). The items were translated to Polish and back-translated by two independent translators. The scale is comprised of three subscales assessing beliefs in the (1) immutability (e.g., “Homosexuality is caused by biological factors such as genes and hormones”), (2) universality (e.g., “Homosexuality has probably existed throughout human history”), and (3) discreteness of homosexuality (e.g., “Homosexuality is a category with clear and sharp boundaries: people are either homosexual or they are not”). The immutability and universality subscales were highly correlated (*r*(877) = .52; *p* < .001) and their correlations with the intuitive disapproval of gay men (immutability: *r*(877) =  − .38; *p* < .001; and universality: *r*(877) =  − .46; *p* < .001) and the IAT score (immutability: *r*(877) =  − .07; *p* = .053; and universality: *r*(877) =  − .11; *p* = .002) were very similar. Thus, based on theoretical considerations the beliefs about genetic bases, immutability and cultural universality as the “born this way” beliefs (Arseneau et al., 2013; Peretz-Lange, 2021) and for the sake of simplicity we collapsed the two scales, creating an index pertaining to the belief in the immutability and universality of homosexuality. Unless otherwise stated, the pattern of the results conducted with the collapsed measure matches those conducted with the independent subscales, and the syntax for these analyses is available along the dataset via OSF (https://osf.io/uzr94). Higher scores indicate stronger endorsement of the essentialist beliefs.

The intuitive disapproval of gay men was assessed by presenting participants with a short vignette describing a movie director who attracted criticism by creating a music videoclip showing two men French kissing in public (Inbar et al., 2009). Participants indicated how much they agreed with following statements: “In my opinion the director intentionally encourages homosexual men to French kiss in public”; “There is something wrong with homosexual men French kissing in public,” and “It is wrong of the director to make a video that encourages homosexual men to French kiss in public.” Attributing intentionality to the director’s choice indicates moral condemnation of the behavior (Inbar et al., 2009). Responses to all three items were highly consistent. We averaged them to a single measure, where higher scores indicate greater moral condemnation of homosexuality.

Implicit homophobia was assessed using the online sexual orientation IAT (Hatzenbuehler et al., 2009; Rowatt et al., 2006). The IAT was programed and administered by the Ariadna Research Panel using stimuli available at https://www.projectimplicit.net/resources/study-materials/ (adapted to Polish by Maison, 2004). The materials detailing the construction of the online IAT by the Ariadna Research Panel and the syntaxes to compute the *d* statistics are available at https://osf.io/uzr94/. Participants followed on-screen instructions. As per the standard IAT procedure, they were asked to categorize stimuli as heterosexual/homosexual and good/bad. Participants were instructed to press the “d” key (on the left side of the keyboard) if the image or word fit the category/ies presented on the left side of the screen and the “k” key (on the right side of the keyboard) if the image or word fit to the category/ies presented on the right side of the screen. If an incorrect key was pressed, participants were asked to correct their response before moving on. The following words were used with positive valence: fantastic, beautiful, love, adore, glorious, cherish, cheer, triumph, and with negative valence: tragic, scorn, yucky, annoy, evil, horrible, hurtful, horrific (in Polish after Maison, 2004; Maison & Mikołajczyk, 2003). Four graphic representations of heterosexual and homosexual couples were used as stimuli, along words representing each category (in Polish): heterosexual, heterosexuality, husband and wife, man and woman for heterosexuality; homosexual, homosexuality, gay(s), lesbian(s) for homosexuality.

Implicit associations are inferred based on a comparison of reaction times when participants make complex categorizations congruent and incongruent with the bias. For example, stimuli are to be categorized as heterosexual or good in categorizations congruent with the bias, or as heterosexual or bad in categorizations incongruent with the bias. Participants classify stimuli faster when making complex categorizations congruent with their bias than when they are incongruent with the bias. The implicit preference for hetero- over homosexuality is expressed by the *d-*score (Greenwald et al., 2003).

In-group satisfaction was assessed using the Polish version of the 4-item in-group satisfaction subscale of the in-group identification scale (as used in previous studies, e.g., “I am glad to be Polish”; Jaworska, 2016; Leach et al., 2008). Higher scores indicate higher in-group satisfaction.

### Results and Discussion

Descriptive statistics and reliabilities for all studies are presented in Table 1. In Study 1, collective narcissism, the belief in the discreteness of homosexuality, the intuitive disapproval of gay men, IAT scores, and in-group satisfaction were positively correlated. The “born this way” index representing the belief in the immutability and universality of homosexuality was negatively associated with these variables. Age was positively associated with the belief in the immutability and universality of homosexuality, the belief in the discreteness of homosexuality, and with scores on the IAT (Table 2). Additionally, men scored significantly higher than women on the intuitive disapproval of gay men (*M*_Men_ = 4.69, SD_Men_ = 1.52; *M*_Women_ = 4.27, SD_Women_ = 1.51; *t*(877) =  − 4.05, *p* < .001*,* Cohen’s *d* =  − .27, 95% CI [− 0.41, − 0.14]). There were no significant differences among men’s and women’s performance on the IAT (*M*_Men_ = .60, SD_Men_ = .35; *M*_Women=_.56, SD_Women_ = .36; *t*(877) =  − 1.69, *p* = .09*,* Cohen’s *d* =  − .11, 95% CI [− 0.09, 0.01]).

**Table 1:** Means, standard deviations, and reliability (Cronbach’s α) for the key variables across the studies

	Study 1			Study 2			Study 3		
	*M*	*SD*	α	*M*	*SD*	α	*M*	*SD*	α
Collective narcissism	3.93	1.37	.91	3.93	1.40	.92	3.98	1.32	.92
Immutability and universality belief	4.80	0.90	.82	4.90	0.92	.81	4.77	1.36	.93
Discreteness belief	3.74	0.96	.68	3.56	0.98	.66	3.67	1.00	.66
Intuitive disapproval of gay men	4.49	1.53	.87	4.38	1.58	0.85	4.62	1.52	.87
Implicit homophobia (*d-*score)	0.58	0.35	–	0.57	0.39	–	0.56	0.39	–
In-group satisfaction	5.24	1.26	.94	5.28	1.15	.92	5.23	1.17	.94

**Table 2:** Correlations among variables in Study 1 (N = 879)

Variables	1.	2.	3.	4.	5.	6.
1. Collective narcissism	–					
2. Immutability & universality	− .42^***^	–				
3. Discreteness	.51^***^	− .45^***^	–			
4. Intuitive disapproval of gay men	.53^***^	− .48^***^	.61^***^	–		
5. IAT	.13^***^	− .10^**^	.17^***^	.18^***^	–	
6. In-group satisfaction	.65^***^	− .19^***^	.28^***^	.33^***^	.07^*^	–
7. Age	− .02	.15^***^	.12^***^	.03	.21^***^	.05

***p ≤ .001. **p < .01. *p < .05

To test H1, predicting that collective narcissism is associated with implicit homophobia, we conducted two linear regressions. We first entered collective narcissism as the predictor, the intuitive disapproval of gay men as the outcome, and controlled for in-group satisfaction, age, and gender (coded 0 = women, 1 = men). The overall model was significant, *F*(4, 874) = 93.03, *p* < .001, *R*^*2*^ = .30. In line with H1, collective narcissism was significantly associated with intuitive disapproval of gay men, *β* = .54, *p* < .001, 95% CI [0.52, 0.69], over and above gender, *β* = .14, *p* < .001, 95% CI [0.24, 0.59], and age, *β* = .01, *p* = .74, 95% CI [− 0.01, 0.01]. In-group satisfaction was not significantly associated with the intuitive disapproval of gay men, *β* =  − .02, *p* = .61, 95% CI [− 0.11, 0.07].

We next ran the same model, entering the IAT’s *d*-scores as the outcome. The overall model was significant, *F*(4, 874) = 14.97, *p* < .001, *R*^*2*^ = .06. In line with H1, collective narcissism was significantly associated with implicit homophobia, *β* = .16, *p* < .001, 95% CI [0.02, 0.06], independently of age, *β* = .22, *p* < .001, 95% CI [0.004, 0.01]. Gender, *β* = .01, *p* = .79, 95% CI [− 0.04, 0.05], and in-group satisfaction, *β* =  − .04, *p* = .31, 95% CI [− 0.04, 0.01], were not related to implicit homophobia. These results corroborate and extend previous findings that Polish collective narcissism is associated with explicit homophobia (Górska & Mikołajczak, 2015; Mole et al., 2022). They suggest that Polish collective narcissism (but not national in-group satisfaction) is also associated with the intuitive moral disapproval of gay men and implicit homophobia.

To test H2, predicting that Polish collective narcissism is associated with implicit homophobia indirectly via the discreteness belief and independently via the “born this way,” immutability and universality beliefs, we conducted two multiple mediation analyses. First, we entered Polish collective narcissism as the predictor, the intuitive disapproval of gay men as the outcome, and the beliefs in the immutability and universality of homosexuality and the belief in the discreteness of homosexuality as independent mediators. We included in-group satisfaction, age, and gender as covariates. We used PROCESS macro for SPSS (Model 4, Hayes, 2018) and asked for 10,000 bootstrapped samples.

**Fig. 1:**
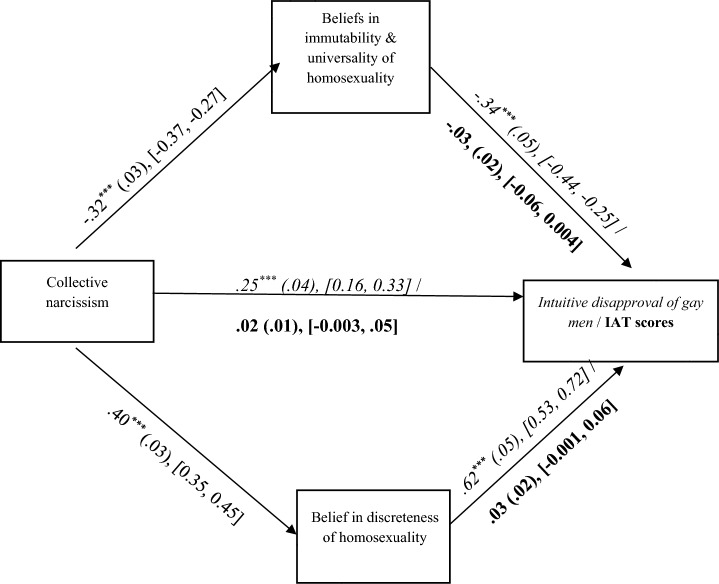
The direct and indirect effects of collective narcissism on the intuitive disapproval of gay men and on IAT scores in Study 1 (N = 879). ***p < .001. 95% CI are in square brackets. The values presented in italics correspond to the analyses conducted on the intuitive disapproval of gay men as the outcome variable. The values presented in bold correspond to the analyses conducted on IAT scores as the outcome variable

The overall model was significant, *F*(6, 872) = 132.04, *p* < .001, *R*^*2*^ = .48. Collective narcissism was negatively associated with the “born this way” beliefs, which in turn were negatively associated with intuitive disapproval of gay men. Independently, collective narcissism was positively associated with the belief in the discreteness of homosexuality, which was, in turn, positively associated with intuitive disapproval of gay men. In line with H2, the indirect association between Polish collective narcissism and the intuitive disapproval of gay men via the “born this way” immutability and universality beliefs, *IE* = 0.11, *SE* = .02, 95% CI [0.07, 0.15], and the indirect association between collective narcissism and the intuitive disapproval of gay men via the discreteness belief, *IE* = 0.25, *SE* = .03, 95% CI [0.20, 0.30] were significant. The direct effect was also significant (Fig. 1).^5^

Next, we tested H2 using the IAT’s *d*-scores as the outcome. The overall model was significant, *F*(6, 872) = 11.59, *p* < .001, *R*^*2*^ = .07. However, neither the beliefs in the immutability and universality of homosexuality, nor in the discreteness of homosexuality were significantly associated with implicit homophobia indicated by the IAT scores. The indirect association between Polish collective narcissism and implicit homophobia via the immutability and universality beliefs, *IE* = 0.01, *SE* = .01, 95% CI [− 0.001, 0.02], and that via the discreteness belief, *IE* = 0.01, *SE* = .01, 95% CI [− 0.001, 0.02] were nonsignificant. The direct effect was also nonsignificant (Fig. 1). These results are at odds with H2. However, the results of the same analysis performed without the covariates (reported in detail in Supplementary Materials) partially support H2: After removing the covariates, the belief in the discreteness of homosexuality predicted IAT scores positively significantly, and the indirect association between collective narcissism and IAT scores via the discreteness belief also turned positive and significant.

The results of Study 1 are in line with and extend the findings that explicit homophobia is positively associated with the belief in the discreteness of homosexuality, but negatively associated with the “born this way” beliefs in the immutability and universality of homosexuality (Haslam & Levy, 2006; Hegarty & Pratto, 2001; Herek & Capitanio, 1995; Huic et al., 2018; Jayaratne et al., 2006; Whitley, 1990). The present results indicate that the same is true for the intuitive disapproval of gay man and that both beliefs mediate the association between Polish collective narcissism and the intuitive disapproval of gay men. Polish collective narcissists endorse the discreteness belief associated with implicit homophobia and do not endorse the “born this way,” immutability and universality beliefs associated with implicit homophobia negatively. However, the results may not generalize across different methods of assessment of implicit homophobia, as the results obtained with the IAT, although in hypothesized directions, were nonsignificant when the analyses were performed with covariates. To provide another test to H1 and H2 to replicate our findings, we recruited a novel sample.

## Study 2

To assure reliability of findings of Study 1, in Study 2, we aimed for their direct replication in a novel sample. We relied on the power analysis conducted for Study 1.

### Method

#### Participants

A representative sample of 388 Polish adults completed the online survey via the Ariadna Research Panel. Participants who took part in Study 1 could not take part in Study 2. Only data from participants who indicated heterosexual orientation were included in the analyses (*N* = 353). We additionally excluded the data of 13 participants who reported technical problems during administration of the IAT and 16 participants who failed to correctly respond to questions checking whether the audio-visual systems in their computers function correctly (necessary for administration of the IAT). The technical check asked participants about the content of short video clips which they were instructed to watch. This was done to make sure participants’ devices are suitable for the IAT to be performed correctly. The survey contained four attention checks as in Study 1. The final sample was made up of 324 participants (175 women, ages 19–76, *M* = 44.19, *SD* = 13.81).

#### Procedure

Participants completed an online survey ostensibly assessing emotions and social attitudes. The procedure contained an experimental manipulation that did not affect the beliefs about homosexuality or the measures of implicit homophobia. The experimental manipulation also did not interact with collective narcissism on key variables of interest: the beliefs about homosexuality, the intuitive disapproval of gay men, and IAT scores. Thus, the data were analyzed cross sectionally. The detailed information about the experimental manipulation and the relevant analyses can be found in Supplementary Materials. Collective narcissism and in-group satisfaction were measured before the manipulation was introduced. The order of the scales and of the items within each scale were presented in a unique randomized order for each participant.

#### Measures

Collective narcissism, the immutability and universality beliefs, the discreteness belief, the intuitive disapproval of gay men, implicit homophobia, and in-group satisfaction were all measured as in Study 1.^6^

### Results and Discussion

In Study 2, collective narcissism, the intuitive disapproval of gay men, and the sexual orientation IAT were positively correlated. The immutability and universality beliefs were negatively associated with each of these variables. The discreteness belief was positively related to collective narcissism, the intuitive disapproval of gay men, in-group satisfaction, age, and negatively to the belief in the immutability and universality of homosexuality. In-group satisfaction was positively related to collective narcissism and the intuitive disapproval of gay men and negatively related to the beliefs in the immutability and universality of homosexuality. Age was positively associated with the beliefs in the immutability and universality of homosexuality and with IAT scores (Table 3). Men scored significantly higher than women on the measure of intuitive disapproval of gay men (*M*_Men_ = 4.69, SD_Men_ = 1.55; *M*_Women=_ 4.11, SD_Women_ = 1.55; *t*(322) =  − 3.35, *p* = .001, Cohen’s *d* =  − 0.37, 95% CI [− 0.59, − 0.15]). IAT scores did not differ across gender (*M*_Men=_ 0.58, SD_Men=_ 0.38; *M*_Women=_ 0.56, SD_Women=_ 0.40; *t*(322) =  − 0.56, *p* = .58, Cohen’s *d* = -0.06, 95% CI [− 0.28, 0.16]).

**Table 3:** correlations among variables in Study 2 (N = 324)

Variables	1.	2.	3.	4.	5.	6.
1. Collective narcissism	–					
2. Immutability & universality	− .35^***^	–				
3. Discreteness	.45^***^	-.39^***^	–			
4. Intuitive disapproval of gay men	.51^***^	− .53^***^	.51^***^	–		
5. IAT	.13^*^	− .12^*^	.10	.19^***^	–	
6. In-group satisfaction	.59^***^	− .24^***^	.13^*^	.29^***^	.04	–
7. Age	− .06	.12^*^	.15^**^	.03	.18^***^	− .04

*p < .05. **p < .01. ***p ≤ .001

To test H1, we conducted two linear regressions entering collective narcissism as the predictor as in Study 1. The overall model for the analyses with the intuitive disapproval of gay men as the outcome was significant, *F*(4, 319) = 32.59, *p* < .001, *R*^*2*^ = .29. In line with H1 and replicating results of Study 1, this analysis yielded a significant association between collective narcissism and the intuitive disapproval of gay men, *β* = .52, *p* < .001, 95% CI [0.45, 0.71], over and above gender, *β* = .18, *p* < .001, 95% CI [0.25, 0.85]. Age, *β* = .03, *p* = .56, 95% CI [− 0.01, 0.01], and in-group satisfaction, *β* =  − .02, *p* = .79, 95% CI [− 0.18, 0.14], did not predict the intuitive disapproval of gay men.

The overall model with the IAT’s *d*-score as the outcome was significant, *F*(4, 319) = 4.61, *p* = .001, *R*^*2*^ = .06. In line with H1 and replicating the findings in Study 1, collective narcissism was significantly associated with the implicit preference for heterosexual over homosexual people, *β* = .17, *p* = .01, 95% CI [0.01, 0.09], over and above age, *β* = .19, *p* < .001, 95% CI [0.002, 0.01]. Gender, *β* =  − .01, *p* = .86, 95% CI [− 0.09, 0.08], and in-group satisfaction, *β* =  − .05, *p* = .42, 95% CI [− 0.06, 0.03], were not related to implicit homophobia.

**Fig. 2:**
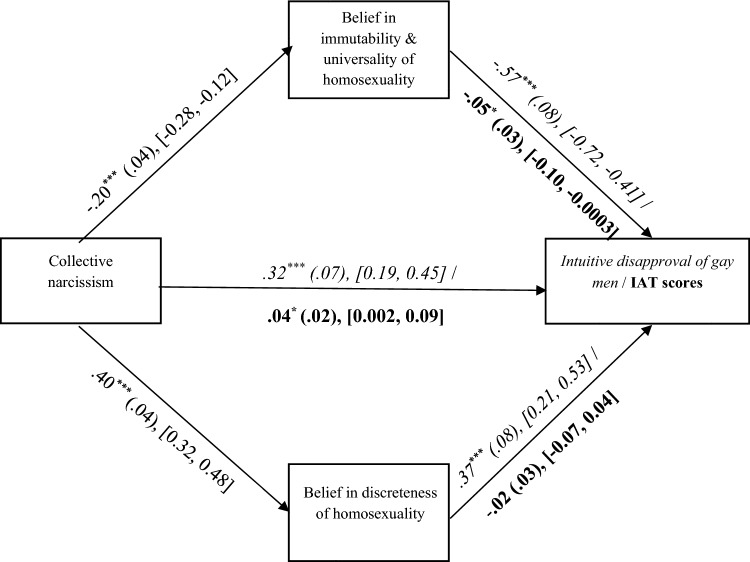
The direct and indirect effects of collective narcissism on the intuitive disapproval of gay men and on implicit homophobia observed in Study 2 (N = 324). *p < .05. **p < .001. 95% CI are in square brackets. The values presented in italics correspond to the analyses conducted on the intuitive disapproval of gay men as the outcome variable. The values presented in bold correspond to the analyses conducted on implicit homophobia operationalized as scores on the IAT as the outcome variable

We tested H2 as in Study 1. The overall model with the intuitive disapproval of gay men as the outcome was significant, *F*(6, 317) = 44.22, *p* < .001, *R*^*2*^ = .46. In line with H2, the indirect association between Polish collective narcissism and the intuitive disapproval of gay men via the beliefs in the immutability and universality of homosexuality was significant, *IE* = 0.11, *SE* = .04, 95% CI [0.05, 0.20]. The indirect effect via the discreteness belief, *IE* = 0.15, *SE* = .05, 95% CI [0.07, 0.26], and the direct effect were also significant (Fig. 2).^7^

The overall model with the IAT’s *d*-score as the outcome was significant, *F*(6, 317) = 3.75, *p* = .001, *R*^*2*^ = .07. Only the immutability and universality beliefs were negatively associated with implicit homophobia measured by the IAT. Note that the overall model related to the association between collective narcissism and the IAT’s *d*-scores was no longer significant after removing the covariates from the model (see Supplementary Materials). In line with H2, the indirect association between Polish collective narcissism and implicit homophobia via the immutability and universality beliefs was significant, IE = 0.01, SE = .01, 95% CI [0.001, 0.01]. Contrary to H2, the indirect association between Polish collective narcissism and implicit homophobia via the discreteness belief was nonsignificant, IE =  − 0.01, SE = .01, 95% CI [− 0.03, 0.02]. The direct effect was significant (Fig. 2).^8^

Thus, in Study 2, we replicated the results supporting H1 and H2 with respect to the intuitive disapproval of gay men in a novel sample. With respect to homophobia assessed by the IAT, we replicated the findings indicating a positive association between Polish collective narcissism and implicit homophobia. In addition, the associations between the essentialist beliefs and implicit homophobia were in the predicted direction, but only the association with the immutability and universality beliefs was significant. The indirect association between collective narcissism and implicit homophobia via this belief was also significant. In sum, the findings suggest that Polish collective narcissism is associated with implicit homophobia assessed as the intuitive moral condemnation of homosexuality and as an automatic negative evaluation of homosexuality and positive evaluation of heterosexuality. The first association is stronger and independently mediated by essentialist beliefs about homosexuality pertaining to structural and agentic explanations of prejudice. The second association is weaker and mediated only by the beliefs regarding the “born this way,” agentic explanation of prejudice, i.e., the immutability and universality beliefs.^9^

## Study 3

In Study 3, we tested H3 and H4, experimentally manipulating the “born this way” belief in the immutability and universality of homosexuality.

### Method

#### Power Analyses

We used G*Power to conduct a priori power calculations (Faul et al., 2007, 2009). We relied on the average effect sizes across social psychological studies (*r* = .21; transformed to *d* = .43 and *f*^*2*^ = .04; Richard et al., 2003). An a priori power analysis for a two-tailed independent samples *t* test revealed that a minimum sample of 172 participants is required to test H3 with alpha level = .05 and power = .80. An a priori power analysis using a linear multiple regression with six predictors revealed that a minimum sample of 347 participants is required to test H4 with alpha level = .05 and power = .80.

#### Participants

Participants were 470 Polish adults, who did not participate in Studies 1 or 2, recruited by the Ariadna Research Panel. The analyses were performed among participants who reported heterosexual orientation (*N* = 426) and correctly responded to an attention check question asking about the content of the article participants read and to four further attention check questions identical to those presented in Studies 1 and 2.^10^ The final sample consisted of 374 participants (161 women, ages 19–80 years, *M* = 45.63, *SD* = 13.92).

#### Procedure

Participants completed an online survey allegedly testing their knowledge about sexuality. First, we administered demographic measures and the measures of Polish collective narcissism and in-group satisfaction. The measures and items were presented in a separate random order, with the order of items separately randomized for each participant.

Next, participants were randomly allocated to one of two research conditions. In the agentic explanation condition (*N* = 186), participants read an alleged report of scientific studies regarding sexual orientations. This report claimed that there is no convincing scientific evidence that sexual orientations are biologically determined, and that they may be shaped by upbringing and the social context. In addition, the report claimed that tolerance toward homosexuality is only present in certain societies at certain times. In the “born this way” condition (*N* = 188), participants read that there is convincing scientific evidence that homosexuality is not a matter of individual choice, that it is biologically determined and cannot be changed. In addition, the report claimed that homosexuality has been present in all societies at all times. Next, we assessed the essentialist beliefs about homosexuality as a manipulation check, the intuitive disapproval of gay men, and administered the sexuality IAT to assess implicit homophobia. Finally, participants were asked to guess the purpose of the experiment (none guessed), debriefed, and thanked.

#### Measures

Collective narcissism, the beliefs in the immutability and universality of homosexuality, the discreteness belief, the intuitive disapproval of gay men, implicit homophobia, and in-group satisfaction were all measured as in Studies 1 and 2.^11^

### Results and Discussion

Collective narcissism, the belief in the discreteness of homosexuality, the intuitive disapproval of gay men, and in-group satisfaction were positively associated. The “born this way” belief was negatively associated with each of these variables, as well as with the IAT scores. The IAT scores were positively associated with the intuitive disapproval of gay men, the belief in the discreteness of homosexuality, and age. Age was positively associated with the belief in the discreteness of homosexuality (Table 4). Men scored higher than women on the intuitive disapproval of gay men (*M*_Men_ = 4.87, SD_Men_ = 1.38; *M*_Women=_4.30, SD_Women_ = 1.64; *t*(372) =  − 3.62, *p* < .001, Cohen’s *d* =  − .38). There were no significant differences between the IAT scores of men (*M*_Men_ = 0.57, SD_Men_ = 0.39) and women (*M*_Women=_0.54, SD_Women_ = 0.38; *t*(372) =  − 0.66, *p* = .51, Cohen’s *d* =  − 0.07, 96% CI [− 0.27, 0.14]).

**Table 4:** Correlations among variables in Study 3 (N = 374)

Variables	1.	2.	3.	4.	5.	6.
1. Collective narcissism	–					
2. Immutability & universality	− .35^***^	–				
3. Discreteness	.40^***^	− .44^***^	–			
4. Intuitive disapproval of gay men	.45^***^	− .46^***^	.58^***^	–		
5. IAT	− .03	− .15^**^	.17^**^	.11^*^	–	
6. In-group satisfaction	.67^***^	− .23^***^	.18^***^	.24^***^	.02	–
7. Age	− .09	.04	.12^*^	− .02	.21^***^	− .01

^*^p < .05. ^**^p < .01. ^***^p ≤ .001

#### Manipulation Check

To check the effectiveness of the experimental manipulation, we conducted an independent samples *t* test entering the “born this way” immutability and universality beliefs as the outcome variable. Levene’s test of equality of error variances was significant, *p* = .03; we thus report the results adjusted for violating this assumption. Participants in the experimental condition (coded as 1) indicated significantly greater beliefs in immutability and universality (*M* = 5.46, SD = 1.03) than those allocated to the control condition (coded as 0; *M* = 4.07, SD = 1.29; *t*(352.75) =  − 11.48, *p* < .001, Cohen’s *d* =  − 1.19, 95% CI [− 1.63, − 1.15]). The manipulation did not affect participants’ belief in the discreteness of homosexuality (*M*_*experimental*_ = 3.61, SD_*experimental*_ = 0.98, *M*_*control*_ = 3.73, SD_*control*_ = 1.02; *t*(372) = 1.08, *p* = .28, Cohen’s *d* = 0.11, 95% CI [− 0.09, 0.32]). These results indicate that the experimental manipulation worked as intended.

#### The Effect on Implicit Homophobia

To test H3, we conducted two independent samples t tests, first with the intuitive disapproval of gay men as the dependent variable and next with the IAT assessed implicit homophobia as the dependent variable. The results revealed that the manipulation did not affect participants’ intuitive disapproval of gay men (*M*_*experimental*_ = 4.57, SD_*experimental*_ = 1.47, *M*_*control*_ = 4.68, SD_*control*_ = 1.58; *t*(372) = 0.73, *p* = .46, Cohen’s *d* = 0.08, 95% CI [− 0.19, 0.43]). The difference between the mean scores was in the expected direction, but it was nonsignificant. However, participants in the experimental condition indicated significantly lower implicit homophobia as assessed by the IAT (*M* = 0.49, SD = 0.41) than those allocated to the control condition (*M* = 0.63, SD = 0.35; *t*(363.27) = 3.57, *p* < .001, Cohen’s *d* = 0.37, 95% CI [0.06, 0.22]). Controlling for collective narcissism did not change the pattern of results (see Supplementary Materials).

To test H4, we conducted two moderation analyses. We first entered the intuitive disapproval of gay men as the outcome, research condition, Polish collective narcissism, and their interaction as predictors. We included age, gender, and national in-group satisfaction as covariates in the model. The overall model was significant, *F*(6, 367) = 19.30, *p* < 001, *R*^*2*^ = .24. Contrary to H4, only collective narcissism, *b* = .63, SE = .09, *p* < .001, 95% CI [0.45, 0.80], and gender predicted the intuitive disapproval of gay men significantly, *b* = .48, SE = .15, *p* = .002, 95% CI [0.18, 0.78]. Consistent with analyses that did not support H3, neither the research condition, *b* = .29, SE = .44, *p* = .51, 95% CI [− 0.58, 1.16], nor its interaction with collective narcissism, *b* =  − .11, *SE* = .11, 95% CI [− 0.31, 0.10], *F*(1, 367) = 1.04, *p* = .31, *R*^*2*^ change = .002 were significant predictors of intuitive disapproval of gay men. The results do not support H4.

We next conducted the same model entering IAT scores as the outcome variable. The overall model was significant, *F*(6, 367) = 5.49, *p* < .001, *R*^*2*^ =. 08. Contrary to H4, only age predicted implicit homophobia significantly, *b* = .01, SE = .002, *p* < .001, 95% CI [0.003, 0.01]. Neither collective narcissism, *b* = .0002, SE = .02, *p* = .99, 95% CI [-0.05, 0.05], the research condition, *b* =  − 0.4, *SE* = 0.12, *p* = .76, 95% CI [− 0.28, 0.20], nor their interaction, *b* =  − .03, *SE* = .03, 95% CI [− 0.08, 0.03], *F*(1, 367) = 0.79, *p* = .37, *R*^*2*^ change = .002, predicted implicit homophobia. Thus, the results do not support H4. The effect of the experimental manipulation on implicit homophobia was not qualified by collective narcissism.

## General Discussion

We investigated the association between Polish collective narcissism and latent, implicit homophobia across two methods of its assessment: the intuitive moral disapproval of gay men and automatic preference for hetero- over homosexuality as assessed by the IAT (Greenwald et al., 1998; Hatzenbuehler et al., 2009; Jost, 2019). We predicted that Polish collective narcissism will be associated with implicit homophobia (H1) and that this relationship will be mediated by the structural (discreteness, positively) and agentic (“born this way,” negatively) beliefs about homosexuality (H2; Haslam & Levy, 2006; Peretz-Lange, 2021). We also predicted that an experimental manipulation that discounts the agentic explanation of homophobia will reduce implicit homophobia (H3), at least on low levels of collective narcissism (H4).

### Summary of Findings

Our results were consistent with H1 and H2 for intuitive disapproval of gay men and less consistently for implicit preference for heterosexuality over homosexuality. The correlation between the two forms of assessment of implicit homophobia is positive and significant but small. Polish collective narcissism is associated with the intuitive disapproval of gay men via the belief that gay people are essentially different than heterosexuals and via the rejection of the belief that they are “born this way.” Implicit negative evaluation of homosexuality as measured by the IAT is associated with Polish collective narcissism via the discounting of the “born this way” beliefs in the immutability and universality of homosexuality but not by the discreteness beliefs. Thus, Polish collective narcissism is associated with implicit homophobia (across measurements) via endorsing the agentic explanation of homosexuality, i.e., attributing homosexuality to individual choice not a genetically based, immutable and culturally universal alternative sexual orientation.

The results of Study 3 were consistent with H3 only for implicit homophobia assessed by the IAT. The “born this way” framing of homosexuality works to reduce implicit negative evaluation of homosexuality relative to heterosexuality (but not to reduce intuitive disapproval of gay men). Our results do not support H4. Contrary to our predictions, the effects of the manipulation of the “born this way” framing of homosexuality were the same on low and high levels of collective narcissism. Thus, Polish collective narcissism was not a barrier for this manipulation to reduce the implicit negative evaluation of homosexual couples in comparison with heterosexual couples.

### Polish Collective Narcissism, Beliefs About Homosexuality, and Implicit Homophobia

Results linking Polish collective narcissism and implicit homophobia extend the previous findings pointing to the robust association between collective narcissism and overt out-group derogation, and prejudice toward stigmatized sub-groups within the nation (Golec de Zavala, 2023; Golec de Zavala & Lantos, 2020; Golec de Zavala et al., 2013, 2019, 2020; Lantos & Forgas, 2021), and specifically toward the LGBT community (Golec de Zavala et al., 2021a, 2021b; Górska & Mikołajczak, 2015; Mole et al., 2022). The present results go beyond those findings indicating that Polish collective narcissism predicts a more subtle and less controllable form of prejudice: implicit homophobia. As such, the present results open a new area for investigation to assess the generalizability of the association between collective narcissism and implicit prejudice across different targets of prejudice and different forms of assessment.

The present results are in line with and extend the literature on essentialist beliefs about homosexuality and homophobia (Arseneau et al., 2013; Haslam & Levy, 2006; Hegarty & Pratto, 2001; Herek & Capitanio, 1995; Huic et al., 2018; Jayaratne et al., 2006; Peretz-Lange, 2021; Whitley, 1990). They indicate that the intuitive moral disapproval of gay men (but not the implicit negative evaluation of homosexuality in comparison with heterosexuality) is linked to essentialist beliefs providing structural explanation for homophobia, pertaining to the belief in essential differences and ingrained hierarchy between hetero- and homosexuals. In contrast, the opposite role of the beliefs discounting individual agency in choosing sexual orientation generalizes across different forms of assessment of implicit homophobia. Endorsing the agentic (“they chose to be this way”) explanation of homosexuality is associated with implicit homophobia assessed as intuitive moral disapproval of gay men and as automatic more positive evaluation of heterosexual over homosexual couples.

The present results afford valuable new insights into the specific beliefs that drive the associations between Polish collective narcissism and implicit homophobia. In line with our hypothesis, cross-sectional analyses in all studies indicate that Polish collective narcissism is associated with supporting structural (“they are different”) and agentic (“they chose to”) beliefs about homosexuality. Polish collective narcissism predicts implicit homophobia predominantly because it is associated with discounting the immutability and universality of homosexuality, the “born this way” belief. According to the attribution theory, when stigma is seen as outside of the individual’s control, the individual is no longer blamed for it, which leads to a decrease in prejudice (Weiner et al., 1988; Whitley, 1990). On the contrary, when one has the ability to choose a social category and behaviors that are viewed as morally wrong, condemned and undesirable, that individual is automatically evaluated negatively (Peretz-Lange, 2021). The “born this way” argument removes homosexuality from the moral domain, as it is no longer a matter of free will and individual choice. In order to morally condemn homosexuality, collective narcissists need to believe that sexual orientation is a matter of choice and human evil design. The present results align with findings suggesting that collective narcissism is associated with the re-framing and justification of discrimination as protection of the in-group (Golec de Zavala et al., 2009, 2016, 2019, 2022). If sexual orientation is a matter of choice than sexual minorities can be framed as choosing to undermine the greatness and purity of the national in-group. Homophobia may be thus re-framed as patriotic protection of the nation from moral contamination.

### The “Born This Way” Belief and a Decrease in Implicit Homophobia

In Study 3, we experimentally induced the beliefs in the immutability and universality of homosexuality vs. the belief in the agentic explanation of homophobia that contradicts it. Experimentally decreasing the agency beliefs regarding homosexuality decreased the automatic negative evaluation of homosexuality assessed by the IAT, but produced a much smaller and statistically not significant change in the intuitive disapproval of gay men (although notably the results were in the anticipated direction). The experimental manipulation of the agency beliefs did not affect the discreteness belief. Those results suggest that the two forms of implicit homophobia assessed in our studies are likely driven by different psychological mechanisms. The different associations of the intuitive moral disapproval of gay men (predominantly with the discreteness belief) and the automatic negative evaluation of homo- relative to heterosexuality (predominantly with the agency beliefs) with essentialist beliefs about homosexuality align with this conclusion.

The intuitive disapproval of gay men is assessed using a self-report questionnaire (Inbar et al., 2009). Although the items of this questionnaire do not directly address participants’ attitudes toward homosexuality, there is nevertheless room for participants to consciously alter their intuitive responses if desired. Moral indignation requires a degree of salience of participants’ moral intuitions that condemn non-normative sexuality. In contrast, the sexual orientation IAT relies fully on participants’ automatic associations assessed by reaction times. Those associations are conditioned during socialization. The possibility to consciously control automatic associations is limited and requires training. In addition, arguably implicit attitudes are less available in introspection and can be at odds with explicitly expressed ones (Greenwald et al., 2002; Jost, 2019).

In sum, our results indicate that attributing agency and choice are important in forming implicit negative evaluation of homosexuality. Perceiving the stigmatized group as profoundly different and inferior is more important to produce moral indignation with homosexuality.

### Limitations and Future Directions

Despite advancing our understanding of the association between Polish collective narcissism and homophobia, the present research is not without limitations that should be taken into account when interpreting the findings. Firstly, we do not know whether the associations between national collective narcissism, essentialist beliefs about homosexuality, and implicit homophobia generalize beyond Poland, where the studies were conducted. There is, however, some evidence that they do. National collective narcissism is related to support for populism across countries (Forgas & Lantos, 2020, 2021), and support for populism is related to homophobia (Russell, 2019; Yatsyk, 2020). Future studies would do well to investigate the indirect associations between national collective narcissism and implicit homophobia in different national contexts.

In addition, our experimental manipulation did not have a neutral condition in which no judgment about agency in homosexuality was made. Thus, we cannot be sure whether encouraging the belief in agency in homosexuality increased implicit homophobia or discounting this belief decreased implicit homophobia. We can only observe the different effects either increasing or decreasing this belief. Future studies would do well to clarify this, comparing both of the experimental conditions employed here to a neutral condition. Future studies should also control potentially confounding variables such as familiarity with members of sexual minority groups and having positive interactions with members of sexual minority groups should be explored that were not controlled in our studies (Górska et al., 2017; Herek & Capitanio, 1996).

Finally, we should note that the IAT paradigm has been criticized by several scholars over the past two decades. Key critiques were based on findings suggesting that the IAT may not predict behavior well (Forscher et al., 2019; Oswald et al., 2013), that uncontrolled factors related to the paradigm may be driving any findings (Fiedler et al., 2006), and on the IAT’s psychometric foundations (Blanton et al., 2006, 2007). On the other hand, prominent scholar across the world also made well-founded arguments for the utility of the IAT, for example, through empirically demonstrating the IAT’s convergent and discriminant validity (Gawronski, 2002), suggesting that the criticisms are based on psychometric assumptions which are misunderstood and unjustified (Nosek & Sriram, 2007), or on subjective ideological factors (Jost, 2019). While attempting to argue for or against the IAT’s value is beyond the scope of the present manuscript, we aimed to acknowledge the potential issues related to the IAT by introducing an alternative measure of the automatic evaluation of gay people, and replicating our results using this measure as well.

## Supplementary Information

Below is the link to the electronic supplementary material.

Supplementary file1 (DOCX 73 KB)

## Data Availability

All datasets can be found on OSF: https://osf.io/uzr94/.

## Footnotes

1 The Rainbow Index ranks European countries on the extent to which their LGBT citizens enjoy legal equality, specifically on the basis of laws and policies that have a direct impact on LGBT people’s human rights in seven categories: equality and non-discrimination; family; hate crime and hate speech; legal gender recognition; bodily integrity; civil society space; and asylum.

2 We acknowledge that the concept of “homophobia”—commonly understood as the “irrational fear or intolerance of homosexuality or homosexual persons” (Herek, 1986) —is controversial as it psychologizes prejudice against homosexual people in the sense that “an irrational fear” is not the fault of the person exhibiting the phobic reaction. In addition, phobias are generally understood to be individual phenomena, whereas homophobia can be promoted by groups and governments, as is the case in Poland. In this article, we use “homophobia” in a broad sense to refer to negative attitudes toward sexual minorities.

3 While the specific focus of our research is gay men, we refer to the broader LGBT community when discussing social attitudes toward both sexual and gender minorities in Poland. This is not to suggest that our findings can necessarily be applied to lesbians, bisexual and trans* individuals. We use the term “homosexual” when referring to legal or political debates or if this is the term used in scientific publications we cite.

4 To be sure, essentialist beliefs about sexual identity are lay theories, not supported by scientific evidence. Research suggests that sexual orientations are fluid rather than fixed (Diamond & Rosky, 2016). In addition, attributing genetic bases to non-normative sexualities may lead to viewing LGBT people as genetically defective and to advocating medical treatment to non-heteronormative behaviors (Hegarty, 2002) or to disadvantaging people who experience their sexual orientations as fluid (Diamond & Rosky, 2016).

5 The pattern of these results largely replicates when entering the immutability and universality beliefs as independent mediators rather than one collapsed variable, however, note that the indirect effect in that case is only significant via the belief in the immutability of homosexuality, and not via the belief in the universality of homosexuality. While collective narcissism was associated with both beliefs, only the immutability belief was associated with the intuitive disapproval of gay men. The syntax for this analysis is available along the dataset via OSF (https://osf.io/uzr94).

6 The immutability and universality subscales were highly correlated (*r*(351) = .57; *p* < .001) and their correlations with the intuitive disapproval of gay men (immutability: *r*(351) =  − .40; *p* < .001; and universality: *r*(351) = −.55; *p* < .001) and the IAT score (immutability: *r*(351) =  −.06; *p* = .28; and universality: *r*(351) =  −. 12; *p* = .03) were very similar.

7 The pattern of these results largely replicates when entering the immutability and universality beliefs as independent mediators rather than one collapsed variable. However, note that when covariates are also included in that model, the indirect effect is only significant via the belief in the universality of homosexuality, and not via the belief in the immutability of homosexuality. Although the association between collective narcissism and the belief in the immutability in homosexuality was significant as well as the association between the immutability belief and the intuitive disapproval of gay men, the indirect association was nonsignificant. When no covariates are included, the results replicate as reported in the manuscript. The syntax for this analysis is available along the dataset via OSF (https://osf.io/uzr94).

8 Note that when entering the immutability and universality beliefs as independent mediators rather than one collapsed variable, neither the direct effect, nor any of the indirect effects are significant in the model. The syntax for the analysis is available along the dataset via OSF (https://osf.io/uzr94).

9 The correlations among the IAT assessment of implicit homophobia, collective narcissism, discreteness and immutability and universality beliefs were not consistent across the two studies. We thus conducted a meta-analytic summary of these results, detailed in Supplementary Materials. Overall, these meta-analytic results support the predicted positive association between national collective narcissism and IAT scores, between the discreteness belief and IAT scores, and the predicted negative association between the immutability and universality beliefs and IAT scores.

10 After reading the report participants answered the attention check question: “The report that you have just read presented scientific evidence that: 1. Sexual orientations are a matter of choice and homosexuality is only present in certain cultures vs. 2. Sexual orientations are genetically determined and present in all cultures and all times.” There were 23 participants who chose response 2 in the control condition and 34 participants who chose option 1 in the experimental condition. We were not able to determine whether the incorrect answers were the consequence of a technical error in survey presentation, coding, or participants erroneously recalling the text. We excluded the data of these participants from the analyses.

11 The immutability and universality subscales were highly correlated (*r*(372) = .80; *p* < .001) and their correlations with the intuitive disapproval of gay men (immutability: *r*(372) =  − .42; *p* < .001; and universality: *r*(372) =  − .45; *p* < .001) and the IAT score (immutability: *r*(372) =  − .13; *p* = .01; and universality: *r*(372) =  − .16; *p* = .002) were similar.
