# Supplementary material for: Born This Way? National Collective Narcissism, Implicit Homophobia, and Homosexual Essentialism in Populist Poland
Source: Arch Sex Behav. 2024 Aug 16;53(10):3907–24. doi: 10.1007/s10508-024-02952-z (PMC11588881; doi:10.1007/s10508-024-02952-z)
Supplement: Supplementary file 1 — Supplementary file1 (DOCX 73 KB) [file 10508_2024_2952_MOESM1_ESM.docx]

**Supplemental Materials**

Born this way? National collective narcissism, implicit homophobia and homosexual essentialism in populist Poland

Dorottya Lantos, Richard C. M. Mole, & Agnieszka Golec de Zavala

Published in *Archives of Sexual Behaviour* in 2024

**Study 1**

**Results**

In the main manuscript, we described the analyses testing H1 and H2 which included covariates. Here, we present the same analyses without the covariates.

**Hypothesis 1**

To test H1, predicting that collective narcissism is associated with implicit homophobia, we conducted two linear regressions. We first entered collective narcissism as the predictor, the intuitive disapproval of gay men as the outcome. The overall model was significant, *F*(1, 877) = 340.32, *p* < .001, *R^2^* = .28. Collective narcissism significantly predicted the intuitive disapproval of gay men, β = .53, *p* < .001, 95% CI [0.53, 0.65]. We next ran the same model, entering the IAT’s *d*-scores as the outcome. The overall model was significant, *F*(1, 877) = 14.96, *p* < .001, *R^2^* = .02. Collective narcissism significantly predicted implicit homophobia, β = .13, *p* < .001, 95% CI [0.02, 0.05].

**Hypothesis 2**

To test H2, predicting that Polish collective narcissism is associated with implicit homophobia indirectly via the discreteness belief and independently via the immutability and universality beliefs, we conducted two multiple mediation analyses. First, we entered Polish collective narcissism as the predictor, the intuitive disapproval of gay men as the outcome, and the beliefs in the immutability and universality of homosexuality and the belief in the discreteness of homosexuality as independent mediators. We used PROCESS macro for SPSS (Model 4, Hayes, 2018) and asked for 10,000 bootstrapped samples.

The overall model was significant, *F*(3, 875) = 260.12, *p* < .001, *R^2^* = .48. Collective narcissism negatively predicted the beliefs in the immutability and universality of homosexuality, which in turn negatively predicted the intuitive disapproval of gay men. Independently, collective narcissism positively predicted the belief in the discreteness of homosexuality, which positively predicted the intuitive disapproval of gay men. In line with H2, the indirect association between Polish collective narcissism and the intuitive disapproval of gay men via the immutability and universality beliefs, *IE* = 0.09, *SE* = .02, 95% CI [0.06, 0.13], and the indirect association between collective narcissism and the intuitive disapproval of gay men via the discreteness belief, *IE* = 0.23, *SE* = .02, 95% CI [0.18, 0.27], were significant. The direct effect was also significant (Figure 1).

Next, we tested H2 using the IAT’s *d*-scores as the outcome. The overall model was significant, *F*(3, 875) = 9.11, *p* < .001, *R^2^* = .03. While the beliefs in the immutability and universality of homosexuality did not predict IAT scores significantly, that in the discreteness of homosexuality did. The indirect association between Polish collective narcissism and implicit homophobia via the immutability and universality beliefs was nonsignificant, *IE* = 0.002, *SE* = .004, 95% CI [-0.01, 0.01], while that via the discreteness belief was positive and significant, *IE* = 0.02, *SE* = .01, 95% CI [0.01, 0.03]. The direct effect was nonsignificant (Figure 1).

**Figure 1**

*The Direct and Indirect Effects of Collective Narcissism on the Intuitive Disapproval of Gay Men and on IAT Scores in Study 1, without Covariates (N = 879)*

Beliefs in immutability & universality of homosexuality

-.27^***^ (.02), [-0.31, -0.23]

*-.35^***^ (.05), [-0.44, -0.25]* /

**-.01, (.02), [-0.04, 0.02]**

*.27^***^ (.03), [0.20, 0.33]* /

**.02 (.01), [-0.003, .05]**

*Intuitive disapproval of gay men* / **IAT scores**

Collective narcissism

*.64^***^ (.05), [0.54, 0.73]* /

**.05^**^ (.01), [0.02, 0.08]**

.35^***^ (.02), [0.32, 0.40]

Belief in discreteness of homosexuality

*Note.* ^***^*p* < .001. ^**^*p* < .01. 95% CI are in square brackets. The values presented in italics correspond to the analyses conducted on the intuitive disapproval of gay men as the outcome variable. The values presented in bold correspond to the analyses conducted on IAT scores as the outcome variable.

**Study 2**

Study 2 included an experimental manipulation of kama muta, the experience of being moved or touched (Zickfeld et al., 2019). Although the experimental manipulation did affect the manipulation check, it had no effect on the variables analyzed as the key outcomes throughout this manuscript. It also did not interact with collective narcissism to predict the essentialist beliefs about homosexuality or implicit homophobia. Here, we describe the experimental manipulation and present its effects on kama muta and on the key outcome variables of this manuscript.

**Procedure**

Participants were randomly allocated to either watch a short video previously found to evoke kama muta (*N* = 162) or one previously found to evoke the emotions of amusement (*N* = 162; e.g., Zickfeld et al., 2019). Both videos are available at via OSF (<https://osf.io/4fha8/>). Following the video, participants answered an attention check question about the content of the video. Sixteen participants responded incorrectly to these, and were thus excluded from the analyses (as disclosed in the Participants section of the main manuscript). Participants were additionally asked whether they had any technical difficulties, and we excluded 13 participants who indicated that they did (as disclosed in the Participants section of the main manuscript). Participants were next presented with manipulation check questions assessing whether they experienced kama muta. The questions used as manipulation check form sections of the Kama Muta Multiplex questionnaire (Zickfeld et. al., 2019, available in Polish translation via OSF at <https://osf.io/cydaw/>).

**Measures**

*Kama muta* was measured using items of the Polish version of the Kama Muta Multiplex Scale (KAMMUS, Zickfeld et al., 2019). Participants responded to all items on a 7-point scale (1 = *completely disagree;* 7 = *completely agree*). Section 1 asked participants to report the extent to which they experienced certain feelings, sensations, or actions (e.g., tears), α *=* .92, *M* = 3.40, *SD* = 1.73. Section 2 consisted of appraisal items (e.g., I observed an exceptional sense of closeness appear), α *=* .96, *M* = 4.29, *SD* = 1.63. Section 3 contained motivational items (e.g., After watching the video, I felt like telling someone how much I care about them), α *=* .95, *M* = 3.51, *SD* = 1.84. Section 4 contained a single item, asking participants to assess the extent to which the statement ‘I felt positive emotions’ is true, *M* = 5.13, *SD* = 1.53. Section 5 asked participants to assess emotion labels with regards to the videos (e.g., It was moving), α *=* .96, *M* = 4.12, *SD* = 2.14. We collapsed scores on these subscales by averaging them to create an overall Kama Muta index, α *=* .96, *M* = 3.83, *SD* = 1.59. For the sake of transparency, we present the results on the index scores of the subscales as well as the collapsed measure.

**Results**

We tested the effects of the experimental manipulation on the manipulation check measures of kama muta, as well as variables of interest: the intuitive disapproval of gay men, IAT scores, the beliefs in the immutability and universality of homophobia, and the belief in the discreteness of homophobia. Collective narcissism and in-group satisfaction were assessed before the manipulation, thus we did not assess the effects of the manipulation on these variables. The results of independent-samples t-tests revealed that the experimental manipulation only affected the manipulation check variables, while it did not significantly affect any of the outcome variables used across the analyses reported in the manuscript (Table 1).

In addition, we tested whether the experimental condition interacted with collective narcissism on key variables of interest: the essentialist beliefs about homosexuality, the intuitive disapproval of gay men, and the IAT scores. We conducted four moderation analyses using Model 1 of PROCESS macro for SPSS (Hayes, 2018). We entered the condition as the predictor, collective narcissism as the moderator, and asked for 10,000 bootstrap samples.

We entered the beliefs in the immutability and universality of homosexuality as the outcome of the first model. The overall model was significant, *F*(3, 384) = 22.73, *p* < .001, *R^2^* = .15. Only collective narcissism was a significant predictor, *b* = -.27, *SE* = .10, *p* = .005, 95% CI [-0.45, -0.08]. Neither the experimental condition, *b* = -.11, *SE* = .26, *p* = .66, 95% CI [-0.63, 0.40], nor its interaction with collective narcissism, *b* = .009, *SE* = .06, 95% CI [-0.11, 0.13], *F*(1, 384) = 0.02, *p* = .89, *R^2^* change < .001, predicted the belief in the immutability and universality of homosexuality.

We next entered the belief in the discreteness of homosexuality as the outcome. The overall model was significant, *F*(3, 384) = 36.53, *p* < .001, *R^2^* = .22. Only collective narcissism was a significant predictor, *b* = .38, *SE* = .10, *p* < .001, 95% CI [0.18, 0.57]. Neither the experimental condition, *b* = .06, *SE* = .27, *p* = .82, 95% CI [-0.46, 0.59], nor its interaction with collective narcissism, *b* = -.03, *SE* = .06, 95% CI [-0.16, 0.10], *F*(1, 384) = 0.22, *p* = .64, *R^2^* change < .001, predicted the belief in the discreteness of homosexuality.

We next entered the intuitive disapproval of gay men as the outcome. The overall model was significant, *F*(3, 384) = 46.43, *p* < .001, *R^2^* = .26. Only collective narcissism was a significant predictor, *b* = .60, *SE* = .16, *p* < .001, 95% CI [0.29, 0.90]. Neither the experimental condition, *b* = .07, *SE* = .42, *p* = .87, 95% CI [-0.76, 0.90], nor its interaction with collective narcissism, *b* = -.002, *SE* = .10, 95% CI [-0.20, 0.20], *F*(1, 384) < 0.001, *p* = .98, *R^2^* change < .001, predicted the belief in the intuitive disapproval of gay men. We finally entered implicit homophobia as the outcome. The overall model was nonsignificant, *F*(3, 384) = 2.28, *p* = .08, *R^2^* = .02, thus, we do not report further results.

**Table 1**

*Means (and Standard Deviations) Corresponding to the Variables Measured in Study 3 Across the Control and Experimental Conditions (N = 324)*

| Measure | Control Condition | Experimental Condition | *t* | *df* | *p* | *d* |
| --- | --- | --- | --- | --- | --- | --- |
| Intuitive disapproval of gay men | 4.42 (1.53) | 4.33 (1.63) | -0.55 | 322 | .58 | -.06 |
| IAT scores | 0.58 (0.36) | 0.55 (0.42) | -0.71 | 322 | .48 | -.08 |
| Immutability & Universality | 4.86 (0.89) | 4.93 (0.96) | 0.71 | 322 | .48 | .002 |
| Discreteness | 3.53 (0.94) | 3.59 (1.01) | 0.57 | 322 | .57 | .08 |
| Kama muta 1 | 2.03 (0.77) | 4.77 (1.28) | 23.29 | 263.26^*^ | < .001 | 2.59 |
| Kama muta 2 | 3.42 (1.54) | 5.15 (1.19) | 11.35 | 302.23^*^ | < .001 | 1.26 |
| Kama muta 3 | 2.36 (1.47) | 4.65 (1.41) | 14.31 | 322 | < .001 | 1.59 |
| Kama muta 4 | 4.35 (1.48) | 5.92 (1.13) | 10.78 | 300.95^*^ | < .001 | 1.20 |
| Kama muta 5 | 2.35 (1.26) | 5.89 (1.14) | 26.54 | 318.93 | < .001 | 2.95 |
| Kama muta collapsed | 2.56 (0.86) | 5.10 (1.06) | 23.65 | 322 | < .001 | 2.63 |

*Note. ^*^*degrees of freedom adjusted as equality of variances assumption was violated.

In the main manuscript, we described the analyses testing H1 and H2 which included covariates. Here, we present the same analyses without the covariates.

**Hypothesis 1**

To test H1, predicting that collective narcissism is associated with implicit homophobia, we conducted two linear regressions. We first entered collective narcissism as the predictor, the intuitive disapproval of gay men as the outcome. The overall model was significant, *F*(1, 322) = 111.03, *p* < .001, *R^2^* = .26. Collective narcissism significantly predicted the intuitive disapproval of gay men, β = .51, *p* < .001, 95% CI [0.46, 0.68]. We next ran the same model, entering the IAT’s *d*-scores as the outcome. The overall model was significant, *F*(1, 322) = 5.48, *p* = .02, *R^2^* = .02. Collective narcissism significantly predicted implicit homophobia, β = .13, *p* = .02, 95% CI [0.01, 0.07].

**Hypothesis 2**

To test H2, predicting that Polish collective narcissism is associated with implicit homophobia indirectly via the discreteness belief and independently via the immutability and universality beliefs, we conducted two multiple mediation analyses. First, we entered Polish collective narcissism as the predictor, the intuitive disapproval of gay men as the outcome, and the beliefs in the immutability and universality of homosexuality and the belief in the discreteness of homosexuality as independent mediators. We used PROCESS macro for SPSS (Model 4, Hayes, 2018) and asked for 10,000 bootstrapped samples.

The overall model was significant, *F*(3, 320) = 85.29, *p* < .001, *R^2^* = .44. Collective narcissism negatively predicted the beliefs in the immutability and universality of homosexuality, which in turn negatively predicted the intuitive disapproval of gay men. Independently, collective narcissism positively predicted the belief in the discreteness of homosexuality, which positively predicted the intuitive disapproval of gay men. In line with H2, the indirect association between Polish collective narcissism and the intuitive disapproval of gay men via the immutability and universality beliefs, *IE* = 0.13, *SE* = .03, 95% CI [0.07, 0.19], and the indirect association between collective narcissism and the intuitive disapproval of gay men via the discreteness belief, *IE* = 0.13, *SE* = .04, 95% CI [0.06, 0.22], were significant. The direct effect was also significant (Figure 2). Next, we tested H2 using the IAT’s *d*-scores as the outcome. The overall model was nonsignificant, *F*(3, 320) = 2.53, *p* = .06, *R^2^* = .02. We thus do not detail these results further.

**Meta-analytic summary**

The correlations among the IAT assessment of implicit homophobia, collective narcissism, discreteness and immutability and universality beliefs were not consistent across the two studies. In order to assess whether the obtained results are reliable, we conducted random effects meta-analysis of those associations using ESCI in Excel for meta-analyses (Cumming & Calin-Jageman, 2017). The Excel files are available via OSF (<https://osf.io/uzr94/>). The combined effect size across Study 1 and Study 2 for the correlations between implicit homophobia measured by the IAT and collective narcissism positive and significant, *r* = .13, 95% CI [.07, .19]. A Diamond ratio of 1.0 indicated low heterogeneity of this association. The combined effect size of the correlations between IAT scores and the ‘born that way’ beliefs in the immutability and universality of homosexuality was negative and significant, *r* = -.11, 95% CI [-.16, -.05]. A Diamond ratio of 1.0 indicated low heterogeneity. The combined effect size of the correlations between the IAT scores and the belief in the discreteness of homosexuality was positive and significant, *r* = .15, 95% CI [.08, .21]. A Diamond ratio of 1.14 indicated low heterogeneity. Overall, these meta-analytic results support the predicted positive association between national collective narcissism and IAT scores, between the discreteness belief and IAT scores, and the predicted negative association between the immutability and universality beliefs and IAT scores.

**Figure 2**

*The Direct and Indirect Effects of Collective Narcissism on the Intuitive Disapproval of Gay Men in Study 2, without Covariates (N = 324)*

Beliefs in immutability & universality of homosexuality

-.23^***^ (.03), [-0.30, -0.16]

-.57^***^ (.08), [-0.72, -0.41]

.31^***^ (.05), [0.21, 0.42]

Intuitive disapproval of gay men

Collective narcissism

.41^***^ (.08), [0.26, 0.57]

.32^***^ (.03), [0.25, 0.38]

Belief in discreteness of homosexuality

*Note.* ^***^*p* < .001. ^**^*p* < .01. 95% CI are in square brackets.

**Study 3**

**Results**

In the main manuscript, we described the analyses testing H3 without any covariates. Here, we replicate these analyses controlling for national collective narcissism. We conducted two univariate general linear models. In the first model, we entered the intuitive disapproval of gay men as the outcome, the research condition as the predictor, and collective narcissism as a covariate. The overall model was significant, *F*(2, 371) = 48.41, *p* < .001. While the effects of the condition on the intuitive disapproval of gay men were nonsignificant, *F*(1, 371) = 0.33, *p* = .57, those of collective narcissism were significant, *F*(1, 371) = 96.14, *p* < .001. We entered IAT scores as the outcome in the second model. The overall model was significant, *F*(2, 371) = 6.53, *p* = .002. While the effects of the research condition were significant, *F*(1, 371) = 12.79, *p* < .001, those of collective narcissism were nonsignificant, *F*(1, 371) = 0.38, *p* = .54.

In the main manuscript, we described the analyses testing H4 which included covariates. Here, we present the same analyses without the covariates. To test H4, we conducted two moderation analyses. We first entered the intuitive disapproval of gay men as the outcome, the research condition, Polish collective narcissism, and their interaction as predictors. The overall model was significant, *F*(3, 370) = 32.88, *p* < .001, *R^2^* = .22. Only collective narcissism predicted the intuitive disapproval of gay men significantly, *b* = .59, *SE* = .07, *p* < .001, 95% CI [0.44, 0.73]. Neither the experimental condition, *b* = .47, *SE* = .45, *p* = .30, 95% CI [-0.41, 1.34], nor its interaction with collective narcissism, *b* = -.14, *SE* = .11, 95% CI [-0.35, 0.07], *F*(1, 370) = 1.66, *p* = .20, *R^2^* change = .004, were significant predictors. We next conducted the same model entering IAT scores as the outcome variable. The overall model was significant, *F*(3, 370) = 4.65, *p* = .003, *R^2^* = .04. Neither collective narcissism, *b* = .004, *SE* = .02, *p* = .84, 95% CI [-0.04, 0.04], the experimental condition, *b* = -.03, *SE* = 0.12, *p* = .82, 95% CI [-0.27, 0.22], nor their interaction, *b* = -.03, *SE* = .03, 95% CI [-0.09, 0.03], *F*(1, 370) = 0.90, *p* = .34, *R^2^* change = .002, predicted implicit homophobia.

**References**

Cumming, G., & Calin-Jageman, R. (2017). *Introduction to the new statistics: Estimation, open science, and beyond*. Routledge Taylor & Francis Group.

Hayes, A. F. (2018). *Introduction to mediation, moderation, and conditional process analysis: A regression-based approach* (Second edition). Guilford Press.

Zickfeld, J. H., Schubert, T. W., Seibt, B., Blomster, J. K., Arriaga, P., Basabe, N., Blaut, A., Caballero, A., Carrera, P., Dalgar, I., Ding, Y., Dumont, K., Gaulhofer, V., Gračanin, A., Gyenis, R., Hu, C.-P., Kardum, I., Lazarević, L. B., Mathew, L., … Fiske, A. P. (2019). Kama muta: Conceptualizing and measuring the experience often labelled being moved across 19 nations and 15 languages. *Emotion, 19*(3), 402–424. https://doi.org/10.1037/emo0000450
